# Simultaneous measurement of the antibody responses against SARS-CoV-2 and its multiple variants by a phage display mediated immuno-multiplex quantitative PCR-based assay

**DOI:** 10.3389/fmicb.2022.968036

**Published:** 2022-08-22

**Authors:** Hanyi Chen, Shen Li, Jiali Wang, Siqi He, Dong Wang, Zhaohui Qian, Dandan Hu, Fangfang Qi, Keping Hu, Chenyi Luo, Jianxun Wang

**Affiliations:** ^1^School of Life Sciences, Beijing University of Chinese Medicine, Beijing, China; ^2^School of Basic Medical Sciences, Chengdu University of Traditional Chinese Medicine, Chengdu, China; ^3^NHC Key Laboratory of Systems Biology of Pathogens, Institute of Pathogen Biology, Chinese Academy of Medical Sciences and Peking Union Medical College, Beijing, China; ^4^Guangzhou Women and Children’s Medical Center, Guangzhou Medical University, Guangzhou, China; ^5^Department of Anatomy and Neurobiology, Zhongshan School of Medicine, Sun Yat-sen University, Guangzhou, China; ^6^The Institute of Medicinal Plant Development, Chinese Academy of Medical Sciences and Peking Union Medical College, Beijing, China; ^7^Andes Antibody Technology Hengshui LL Company, Hengshui City, China; ^8^Shenzhen Research Institute, Beijing University of Chinese Medicine, Shenzhen, China

**Keywords:** phage display, coronavirus, SARS-CoV-2, variants, antibody responses

## Abstract

To combat the continued pandemic of COVID-19, multiplex serological assays have been developed to comprehensively monitor the humoral immune response and help to design new vaccination protocols to different SARS-CoV-2 variants. However, multiplex beads and stably transfected cell lines require stringent production and storage conditions, and assays based on flow cytometry is time-consuming and its application is therefore restricted. Here, we describe a phage display system to distinguish the differences of immune response to antigenic domains of multiple SARS-CoV-2 variants simultaneously. Compared with linear peptides, the recombinant antigens displayed on the phage surface have shown some function that requires the correct folding to form a stable structure, and the binding efficiency between the recombinant phage and existing antibodies is reduced by mutations on antigens known to be important for antigen–antibody interaction. By using Phage display mediated immuno-multiplex quantitative PCR (Pi-mqPCR), the binding efficiency between the antibody and antigens of different SARS-CoV-2 variants can be measured in one amplification reaction. Overall, these data show that this assay is a valuable tool to evaluate the humoral response to the same antigen of different SARS-CoV-2 variants or antigens of different pathogens. Combined with high-throughput DNA sequencing technology, this phage display system can be further applied in monitoring humoral immune response in a large population before and after vaccination.

## Introduction

Since coronavirus disease-2019 (COVID-19) emerged at the end of 2019, the continued severe acute respiratory syndrome coronavirus-2 (SARS-CoV-2) pandemic has caused a worldwide public health crisis. A member of the β-coronavirus genus, SARS-CoV-2, has a 29,903 bp genome that codes for structural proteins, including envelope (E), spike (S), nucleocapsid (N), and membrane (M), and nonstructural proteins required for virus infection (Khailany et al., 2020). In the process of virus infection, the receptor-binding domain (RBD) of spike can bind angiotensin-converting enzyme 2 (ACE2) and mediate SARS-CoV-2 entry into the cell (Lan et al., 2020). Because of its importance in terms of virus tropism and infectivity, the S protein has become the target of most vaccines and antibody drugs (Barnes et al., 2020). However, as a single-stranded positive-strand RNA virus with a high mutation rate, mutations have accumulated during the SARS-CoV-2 pandemic, and the variants with increased fitness and potential to escape the immune response have developed, increasing the chance of spread (Harvey et al., 2020).

Since it was first reported in January 2020, the variant with the D614G mutation has replaced the wild type and become the mainstream variant worldwide. Many studies have shown that D614G can significantly enhance the infectivity of SARS-CoV-2, but this mutation does not affect the neutralizing effect of monoclonal antibodies (Yurkovetskiy et al., 2020). At the end of 2020, the variant B.1.351 (Beta) was first reported in South Africa and repeatedly connected with immune escape. B.1.351 has three mutations in the RBD region, K417N, E484K, and N501Y. Some research has shown that E484K and N501Y change the spatial structure of the RBD and reduce the binding efficiency of existing antibodies (Weisblum et al., 2020). B.1.617.2 (Delta) was first discovered in India and became a mainstream variant in 2021. The mutations L452R and T478K in the RBD region may help the virus resist neutralization (Wall et al., 2021). In addition, the P681R mutation improves the replication efficiency of the virus in the human airway system (Saito et al., 2022). In November 2021, variant B.1.1.529 was named Omicron, which overtook the Delta strain in a short time and currently dominates globally. The Omicron variant has more than 50 mutations, 30 of which are located in spike. Because of its enhanced transmissibility and immune evasion capability, Omicron has caused unprecedented concerns worldwide (Callaway, 2021). In the context of increasing vaccination rates and the emergence of variants, evaluating the humoral immune status in a human population toward the different variants and adjusting the countermeasures will play an essential role in counteracting the spread of the virus.

Serological assays have been performed on linear peptides or full-length antigen proteins, and their binding activity has been observed with antibodies. Since the COVID-19 pandemic started, many serological assays based on the spike, nucleocapsid, and other proteins of SARS-CoV-2 have been developed (Torres et al., 2019; Candel et al., 2020; Zhao et al., 2020). These assays employ different techniques, such as ELISA, lateral flow immunoassay (LFIA), and chemiluminescence enzyme immunoassay (CLIA). However, most of these techniques detect only the antibody level to a certain kind of protein in a test. Based on fluorescence immunoassays, Luminex can assay the presence or absence of antibodies to three different SARS-CoV-2 antigens, such as S1, the RBD, and nucleocapsid (xMAP®, 2022). In 2021, Niklas et al. transfected wild-type (WT) or mutant S proteins into the Ramos human B lymphoma cell line and used a color-based barcoded spike flow cytometric assay (BSFA), which allows comparison of the level of antibodies to the S protein of WT SARS-CoV-2 and variants (Vesper et al., 2021). However, multiplex beads and stably transfected cell lines which used in fluorescence immunoassays require stringent production and storage conditions, and assays based on flow cytometry will still be restricted by throughput. Peptide microarrays can immobilize short peptides on solid planar supports and detect different pathogen-related peptides or epitopes in high throughput (Wang et al., 2020; Vengesai et al., 2022). Based on recombinant antigens, protein microarray can be also used to assay the antibody response to different antigens of SARS-COV-2. But considering the high cost of a commercial protein microarray, this method will still be limited in large population assay (Bostan et al., 2020; Krishnamurthy et al., 2020; Hein et al., 2022). Phage immunoprecipitation sequencing (PhIP-Seq) was first reported in 2011, and phage-displayed antigen libraries are encoded by synthetic oligonucleotides. After immunoprecipitation with serum samples, deep DNA sequencing can permit the quantification of each peptide’s antibody-dependent enrichment (Larman et al., 2011). In this way, humoral immune assays can utilize to DNA sequencing, significantly improving the throughput of the assay (Table 1). However, because of the limited length of the synthetic oligonucleotide library, this phage display method can detect only linear epitope-directed antibodies (Mohan et al., 2018). Stoddard et al. (2021) captured immunogenic peptides spanning the entire proteome of SARS-CoV-2 in a phage-displayed antigen library. Through the humoral immune assays of 19 COVID-19 patients, S, N, and ORF1ab were identified as highly immunogenic regions. However, due to the conformational tendency, this study did not identify any antibody-dependent enrichment in the RBD.

**Table 1:** The throughput of common serological assays to SARS-CoV-2.

Test type	Throughput
ELISA	Single detection
Lateral flow immunoassay (LFIA)	Single detection
Chemiluminescence enzyme immunoassay (CLIA)	Single detection
Fluorescence immunoassays (FIA)	Multiple assays
Peptide microarrays	High-throughput assay
PhIP-Seq	High-throughput assay

**Figure 1:**
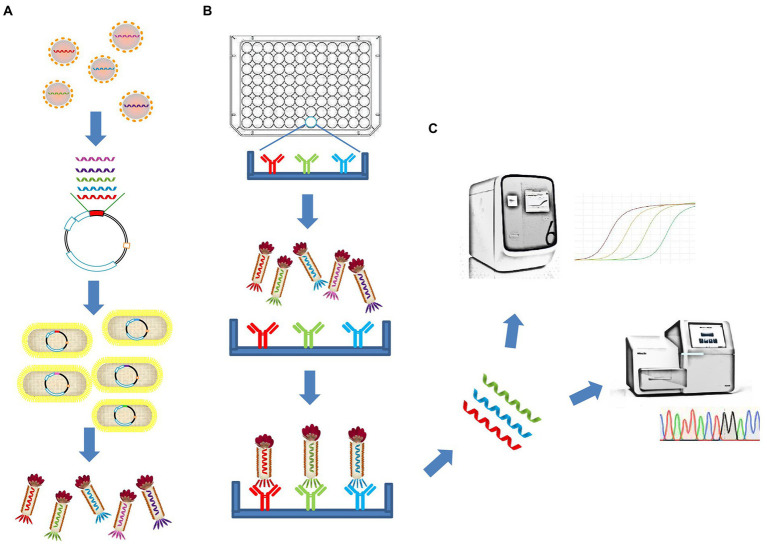
Schematic illustration of the polyvalent phage display system and the Pi-mqPCR assay. **(A)** Construction and synthesis of the recombinant phage. **(B)** Phage immunoprecipitation. **(C)** Real-time immuno-PCR and high-throughput DNA sequencing assay.

Here, we describe a polyvalent phage display system based on the fusion of the entire RBD of SARS-CoV-2 with the M13 bacteriophage Protein III and display it on the phage surface. In addition to detecting linear epitope-directed antibodies, the recombinant antigens displayed on the phage surface has shown some function that requires the correct folding to form a stable structure compared with linear peptides, including reduced binding efficiency of existing antibodies by mutations and binding with receptor ACE2. By using Phage display mediated immuno-multiplex quantitative PCR (Pi-mqPCR), the binding efficiency between the antibody and different SARS-CoV-2 variants was compared in the same amplified reaction. In this way, antigen–antibody reaction can be turned into DNA assays and significantly improve the throughput of the assay (Figure 1).

## Materials and methods

### Plasmids and bacterial strains

The plasmid pUC19 containing the codon-optimized genes for the SARS-CoV-2 spike RBD (WT), spike NTD, nucleocapsid protein, and hemagglutinin HA1 subunit was commercially synthesized (GENERAL BLOT). The gene for the SARS-CoV-2 spike RBD (Omicron) was a gift from Dr. Zhaohui Qian. The protein sequences used for our phage display system were from the SARS-CoV-2 Wuhan strain (MN908947), SARS-CoV-2 Omicron strain (R40B60 BHP 3321001247/2021), influenza virus A/Perth/16/2009(H3N2; KM821346), and A/WSN/1933(H1N1; HE802059). M13KO7 helper phage was provided by New England Biolabs. Competent *Escherichia coli* TG1 and DH5α cells were obtained from Biomed.

### Construction of the recombinant phage

The M13KO7 helper phage was used to infect a culture of TG1 cells. After growth overnight at 37°C, the TIANprep Mini Plasmid Kit (TIANGEN) was used to extract the double-stranded DNA (dsDNA) phage chromosome. The insert genes and the M13KO7 phage chromosome were amplified by PCR with primers containing 20 base pair overlap. The PCR products were assembled by an EasyGeno Assembly Cloning Kit (TIANGEN) at 50°C for 30 min. The assembly mix was transformed into competent DH5α cells. The point mutations in the RBD region were generated by QuikChange Lightning (Agilent). The recombinant phage chromosome was sequenced (GENEWIZ), and the sequencing results were analyzed by SnapGene 4.2 software. To produce the recombinant phage, the DH5α strain transformed with the recombinant phage chromosome was grown overnight at 37°C and centrifuged for 15 min at 8,000 × rpm at 4°C. The supernatant was collected, and the recombinant phage particles were precipitated by adding 20% (w/v) polyethylene glycol (PEG) 8,000 solution to 2.5 M NaCl (LABLEAD) in distilled water. Following incubation on ice for 1 h, the mixture was centrifuged for 30 min at 10,000 × rpm at 4°C, and the pellet was resuspended in 1 ml of PBS and stored at 4°C. After incubation at 95°C for 15 min, real-time fluorescent quantitative PCR was used to detect the titer of the phage.

### Western blot assay

Before denaturation at 99°C for 20 min, 10 μl of 5× SDS–PAGE Sample Loading Buffer (LABLEAD) was added to 40 μl of phage (109 copies/μl). Then, the samples were electrophoresed on LabPAGE 4–12% 11-well gels (LABLEAD) and transferred to a nitrocellulose membrane (APPLYGEN). The nitrocellulose membrane was blocked with 5% skim milk for 2 h, after which the anti-myc tag antibody (R&D Systems) was added and incubated overnight at 4°C. The membrane was washed three times with TBST (LABLEAD) and then incubated with a horseradish peroxidase-labeled goat anti-mouse IgG secondary antibody (1:5000, APPLYGEN) at room temperature for 1 h. After washing the membrane three times with TBST, SuperSignal West Pico PLUS Chemiluminescent Substrate (Thermo Fisher) was added, and the ChemiDoc MP Imaging System (Bio-Rad) was used to record images.

### Phage immunoprecipitation

The binding protein was coated in a 96-well microplate with 50 μl of ELISA Coating Buffer (Solarbio) and incubated at 4°C overnight. After washing five times with PBST (LABLEAD), blocking was performed with a 2% BSA (LABLEAD) solution in PBST for 2 h at 37°C. Following further washing with PBST four times, 50 μl/well recombinant phage (10^11^ copies/ml) was added to the microplate for 1 h at 37°C. After washing five times with PBST, 100 μl of DNase/RNase-free water (Solarbio) was added to each well of the microplate and incubated at 95°C for 20 min. The eluted phage particles were used as DNA templates in real-time quantitative PCR.

### Real-time immuno-PCR assay

For the real-time fluorescent quantitative PCR, 10 μl of 2× Realab Green PCR Fast Mixture [LABLEAD, forward primer (0.5 μM), reverse primer (0.5 μM); Table 2] and 1 μl of eluted phage templates were added to each PCR tube. DNase/RNase-free water was added to bring the up volume to 20 μl. The thermal cycle conditions included 95°C for 30 s, followed by 40 cycles of 95°C for 10 s and 60°C for 30 s. The amplification system of multiplex real-time fluorescent quantitative PCR consisted of 5 μl of 2× FastFire qPCR PreMix (TIANGEN), forward primer (0.3 μM), reverse primer (0.3 μM), four types of probes (0.4 μM each), 0.5 μl of eluted phages, and sterilized water to a final volume of 10 μl. After 1 min of incubation at 95°C, 40 PCR cycles were performed according to the following temperature regime: 95°C for 5 s, 57°C for 10 s, and 72°C for 20 s. The amplification was performed using a Stratagene Mx3005P real-time PCR system (Agilent). The copies of phage chromosomes were determined by plotting Ct versus 10-fold serial dilutions of target gene fragments with known DNA concentrations.

**Table 2:** Primer and probe sequences used in multiplex real-time fluorescent quantitative PCR.

Primer/probe	Sequence (5′–3′)
Forward primer	ACTGCGGCGAGCGGAAAT
Reverse primer	GCCACCACTGATTTGAGCG
Probe-1	FAM-AACAACTGGACCGACCG-BHQ1
Probe-2	CY5-CTGGCTCTGCGTGCTGTGCTC-BHQ2
Probe-3	ROX-CTCAAACCCCCGCGCGTTCCCC-MGB
Probe-4	VIC-TCCAAGCGCTCGCATCGTGG-BHQ1

### Statistical analysis

In this study, statistical analysis was performed by GraphPad Prism 8.0 (GraphPad Software) by using a *t-*test, and *p* < 0.05 was considered significant. Experimental data were expressed as the mean ± standard error of the mean.

## Results

### Construction and synthesis of the recombinant phage

**Figure 2:**
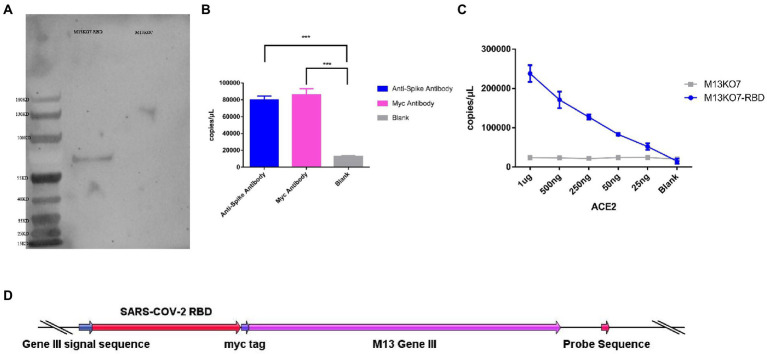
Construction and synthesis of the recombinant phage. **(A)** Detection of the expression of the myc tag on wild-type M13KO7 and the recombinant phage by Western blotting. **(B)** The enrichment of the recombinant phage among the antibodies targeted to the spike protein of SARS-CoV-2 and myc tag; ***p < 0.001. **(C)** The enrichment of the RBD-displaying phage and the wild-type M13KO7 by ACE2. **(D)** The phage vectors used to express the polyvalent display phage. The values shown are the average of three independent experiments and their standard errors.

To construct the phage displaying polyvalence, the RBD-coding gene was inserted into the genome of M13 bacteriophage between the signal sequence and Gene III. A myc tag was used to detect the expression of the recombinant protein (Figure 2D). Western blot results indicated that recombinant protein III migrated at the expected molecular weight of approximately 65 kDa (Ledsgaard et al., 2018; Tai et al., 2020), while Protein III of wild-type M13KO7 could not bind with the anti-myc-tag antibody (Figure 2A).

The binding activity of recombinant phage was evaluated by real-time fluorescent quantitative PCR. After incubation at 95°C for 20 min, both the microplates coated with anti-RBD antibody (Sino Biological, Cat. # 40150-R007, RRID Number: AB_2827979) and anti-myc-tag antibody (APPLYGEN, Cat. # C1302-100) showed significant enrichment of phage DNA (Figure 2B). The result shows that the recombinant protein has displayed on the surface of the phage and could be identified by the antibodies.

### The function of the recombinant RBD constructs

**Figure 3:**
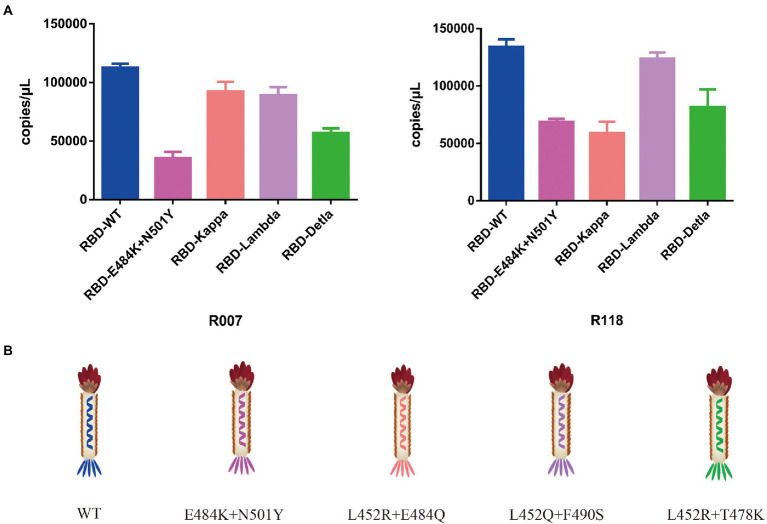
**(A)** The binding activity between the recombinant phage with different mutations in the RBD construct and two commercially available anti-RBD antibodies. **(B)** Schematic drawing of the phage of the phage-displayed RBD construct with different mutations. The values shown are the average of three independent experiments and their standard errors.

To test the function of the recombinant RBD constructs, we coated the recombinant human ACE2 protein in the microplate and assayed the enrichment of recombinant phage and WT M13KO7 after immunoprecipitation. In contrast with M13KO7, quantitative PCR showed that the recombinant phage can bind with human ACE2 protein in a concentration-dependent manner (Figure 2C). Next, we wanted to learn more about how the mutations in the phage-displayed RBD construct influenced the recognition of anti-RBD antibodies. We introduced the RBD-displayed phage with L452R, T478K mutations (B.1.617.2, Delta variant), L452Q, F490S mutations (C37, Lambda variant), L452R, E484Q mutations (B.1.617.1, Kappa variant), and N501Y, E484K mutations, which are characteristic of the Alpha and Beta variants (Figure 3B). After immunoprecipitation with two commercially available anti-RBD antibodies (R007 and R118), quantitative PCR showed that the enrichment of recombinant phage could be reduced by mutations in the RBD construct, especially the L452R, T478K, N501Y, and E484K mutations (Figures 3A,B). These data demonstrate that the recombinant RBD displayed on the surface of the phage has more function which requires the correct folding to form a stable structure than linear peptides (Chen et al., 2020).

### Phage display mediated immuno-multiplex quantitative PCR assay

**Figure 4:**
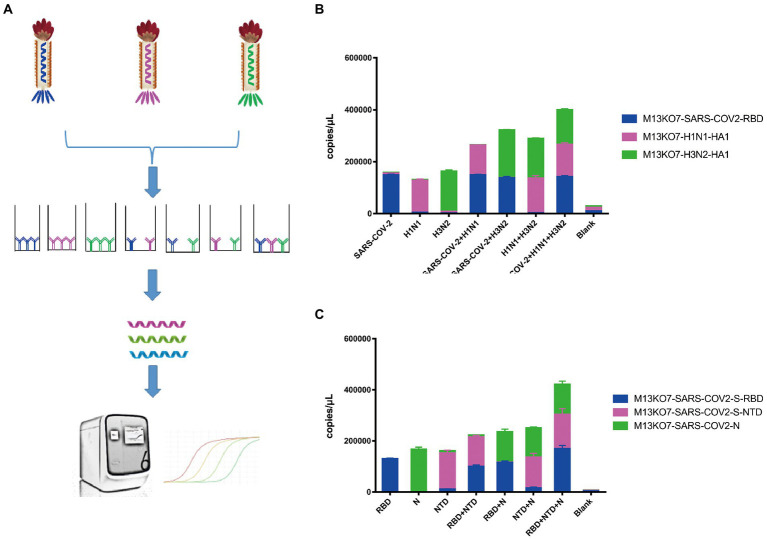
Pi-mqPCR was used to identify the binding specificity of recombinant phage. **(A)** Schematic drawing of the Pi-mqPCR. **(B,C)** Distribution of the phage chromosome that displayed antigens from different viruses **(B)** and different regions of SARS-CoV-2 **(C)** following immunoprecipitation with the respective labeled antibodies. The values shown are the average of three independent experiments and their standard errors.

To directly assay the enrichment of different recombinant phage, a sequence that could be identified by probes was inserted into the phage chromosome (Figure 2D). Based on this system, Pi-mqPCR can compare the binding activity of recombinant phages displaying different antigens through the same amplification reaction. To identify the binding specificity between the recombinant phage and different antibodies, we first constructed a recombinant phage with the N-terminal domain of the S protein and a C-terminal truncated version of the nucleocapsid protein from SARS-CoV-2. In addition, the hemagglutinin HA1 subunit from influenza virus A/Perth/16/2009(H3N2) and A/WSN/1933(H1N1) was also chosen to construct the recombinant phage. After coating the microplate with the corresponding antibodies, different recombinant phages were then equally mixed for immunoprecipitation (Figure 4A). The result of Pi-mqPCR shows that all the types of recombinant phage showed enrichment in only the microplate coated with the respective labeled antibodies. Based on this result, we found that this system can be used to identify different antibodies not only targeting different viruses (Figure 4B) but also targeting different regions of SARS-CoV-2 (Figure 4C).

**Figure 5:**
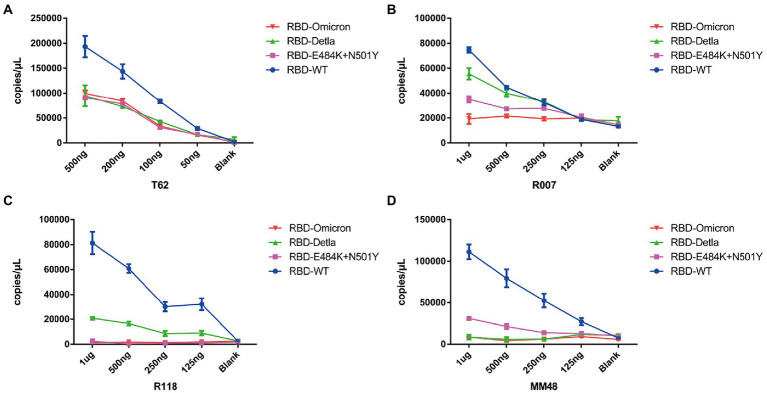
Pi-mqPCR assay to explore the enrichment of the recombinant phage-displayed RBD constructs from wild-type SARS-CoV-2 and different variants by a polyclonal antibody **(A)** and three monoclonal antibodies **(B–D)**. The values shown are the average of three independent experiments and their standard errors.

**Figure 6:**
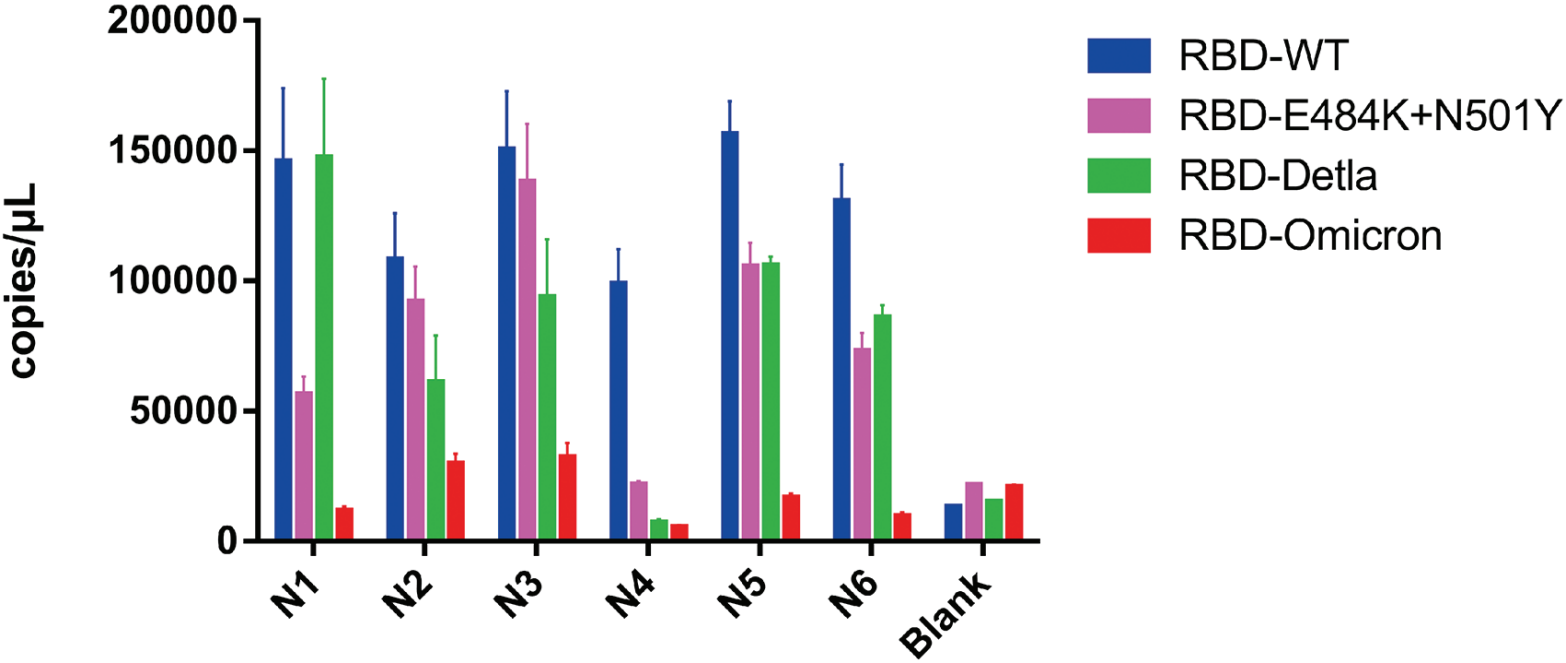
Pi-mqPCR assay to explore the enrichment of the recombinant phage-displayed RBD constructs from wild-type SARS-CoV-2 and different variants by six anti-SARS-CoV-2 RBD nanobodies. The values shown are the average of three independent experiments and their standard errors.

Having demonstrated that Pi-mqPCR system works well in the evaluation of binding specificity, we next used it to identify the phage-displayed RBD constructs from different variants. We observed binding activity between the phage displaying the RBD of the Delta variant (B.1.617.2) and Omicron variant (B.1.1.529) and that with N501Y and E484K mutations and four commercially available anti-RBD antibodies (Sino Biological), including a polyclonal antibody (T62: Cat. # 40591-T62) and three monoclonal antibodies (R007: Cat. # 40150-R007, RRID Number: AB_2827979, R118: Cat. # 40592-R118, and MM48: Cat. # 40591-MM48). In addition, six anti-SARS-CoV-2 RBD nanobodies selected in previous research (N1-N6) were also used for immunoprecipitation with the recombinant phage. We observed that all four types of recombinant phage could bind the polyclonal antibody in a concentration-dependent manner. However, compared with the wild type, the recombinant phage that displayed RBD mutants showed more reduced enrichment (Figure 5A). Interestingly, the titration of the three RBD-specific monoclonal antibodies showed a significant difference in the enrichment between the RBD constructs from wild-type and different SARS-CoV-2 variants. The RBD constructs from the Delta variant could still be recognized by the antibodies R007 and R118 in a concentration-dependent manner. However, the binding activity of the phage-displayed RBD with N501Y and E484K point mutations could be observed only at high concentrations of R007 and MM48, while the RBD region from Omicron showed little enrichment with all three monoclonal antibodies (Figures 5B–D). Similar to the monoclonal antibodies, the six types of anti-SARS-CoV-2 RBD nanobodies showed a greater decline in binding activity with RBD constructs from different variants, especially the Omicron variant (Figure 6). In addition, we compared the binding activity between nanobody N4, N6, and SARS-CoV-2 (2019-nCoV) Spike RBD Recombinant Protein from wild type (Sino Biological, Cat: 40592-V05H) and Omicron variant (Sino Biological, Cat: 40592-V05H3). In line with Pi-mqPCR, the result of the standard ELISA-based method showed that the binding activity between nanobody N4, N6, and RBD Recombinant Protein from Omicron variant reduced significantly compared with the wild type (Supplementary Figure S1). These results are in line with research showing that mutations in the RBD region reduce the binding efficiency of existing antibodies by changing the spatial structure of the RBD (Garcia-Beltran et al., 2021; Li et al., 2021; Liu C, et al., 2021; Liu Z, et al., 2021; Mlcochova et al., 2021), and the Omicron variant shows the most resistance to neutralization by monoclonal and convalescent plasma antibodies (Zhang et al., 2022). Based on these data, the phage-displayed antigen system can be used to evaluate the fine specificity of the antibody response to different SARS-CoV-2 variants.

## Discussion

In this study, we constructed a polyvalent antigen display system based on the M13 bacteriophage. We show here that the recombinant antigen could be displayed on the surface of the phage and identified by antibodies. Interestingly, we observed binding activity between ACE2 and the phage that displayed the RBD region of SARS-CoV-2. These data demonstrated that the phage-displayed antigen has properties similar to the protein found on the viral membrane. Based on this result, the phage-displayed antigen can be used not only to detect linear-directed antibodies but also in some research that requires the correct structure. For example, Uppalapati et al. (2022) reconstituted the receptor-binding motif (RBM) of Middle East respiratory syndrome coronavirus (MERS-CoV) by phage-display conformer libraries and selected two reconstituted RBM conformers that cross-reacted with a panel of 7 neutralizing monoclonal antibodies. Combined with phage display selection, phage display antigen libraries can also be used to predict RBD constructs that have a higher affinity for ACE2.

To isolate biomolecules with high affinity, the dual plasmid helper phage display system has been used for phage display selection (Kumar et al., 2022); compared with this, the polyvalent display system can reduce nonspecific binding and has more advantages in diagnostic assays. In this research, our system could identify antibodies that target more than three antigens in the same multiplex real-time fluorescent quantitative PCR. Several studies have shown that serological assays based on multiple antigens can indeed increase the specificity of testing and provide a more comprehensive picture of the humoral immune response (Gillot et al., 2020). For example, the research of Grossberg et al. (Grossberg et al., 2021) showed that the antibody levels of S1-RBD IgA, NP IgG, and S2 IgA can be used to identify severe, mild, and asymptomatic groups of COVID-19 patients. Considering increasing vaccination rates, combined detection of anti-NP and anti-Spike antibodies can also be used to differentiate the immune response from viral infection and accurately assess immunity (Brochot et al., 2022). Compared with flow cytometry, the assay based on Pi-mqPCR can be combined with nucleic acid amplification tests and is more suitable for clinical serological diagnosis.

Since 2020, many studies have shown that mutations in the RBD region of SARS-CoV-2 can reduce the binding efficiency of monoclonal antibodies and convalescent plasma. However, limited to the linear epitope, there are very few phage-displayed antigen libraries available for differentiating the immune response to SARS-CoV-2 variants (Krishnamurthy et al., 2020; Liu, VanBlargan et al., 2021). In this research, we observed that the RBD region of SARS-CoV-2 displayed on M13 bacteriophage can be identified by antibodies and that mutations in the RBD decrease the binding efficiency. In line with a previous study, the Omicron RBD was mostly unable to bind all the types of monoclonal antibodies and nanobodies, while the RBD constructs from the Delta variant and those with the N501Y and E484K point mutations could still bind with specific antibodies. Based on this result, our system can evaluate the humoral response to different SARS-CoV-2 variants at least to a certain extent. Due to anti-infection measures, the convalescent plasma of COVID-19 patients is unavailable for our research. However, based on the results of this research, short DNA sequences, such as probes used in Pi-mqPCR or synthetic barcodes, can be inserted into the genome of M13 bacteriophage and used to measure the phage quantity. For high-throughput assay, the phage which has special barcodes can be used for immunoprecipitation with sera of the people before and after vaccination. After this, high-throughput DNA sequencing can analyze the enrichment of phage DNA to measure the humoral immune response from individual people samples. So combined with high-throughput DNA sequencing technology, this phage display system can be further applied in monitoring humoral immune response in a large population before and after vaccination. Compared with that of antigen expressed in eukaryotic systems, the dramatically lower manufacturing costs will also expand the application ranges of phage-based immunoassays.

Overall, these data show that this Pi-mqPCR assay is a valuable tool to evaluate the humoral response to the same antigen of different SARS-CoV-2 variants or antigens of different pathogens.

## Data availability statement

The raw data supporting the conclusions of this article will be made available by the authors, without undue reservation.

## Author contributions

JnW conceived and designed the experiment, with help from DW, ZQ, DH, FQ, KH and CL. HC and SL performed experiments and analyzed the data. JlW and SH generated anti-SARS-CoV-2 RBD nanobodies. HC wrote the manuscript, with help from all other authors. All authors contributed to the article and approved the submitted version.

## Funding

JnW was supported by startup funds from Beijing University of Chinese Medicine (Grant No. 1000041510051). DH was supported by a grant from Science and Technology Key Project for People’s Livelihood of Guangzhou, China (Grant No. 202206010060).

## Conflict of interest

KH was employed by Andes Antibody Technology Hengshui LL Company.

The remaining authors declare that the research was conducted in the absence of any commercial or financial relationships that could be construed as a potential conflict of interest.

## Publisher’s note

All claims expressed in this article are solely those of the authors and do not necessarily represent those of their affiliated organizations, or those of the publisher, the editors and the reviewers. Any product that may be evaluated in this article, or claim that may be made by its manufacturer, is not guaranteed or endorsed by the publisher.
