# Supplementary material for: Simultaneous measurement of the antibody responses against SARS-CoV-2 and its multiple variants by a phage display mediated immuno-multiplex quantitative PCR-based assay
Source: Front Microbiol. 2022 Aug 22;13:968036. doi: 10.3389/fmicb.2022.968036 (PMC9441900; doi:10.3389/fmicb.2022.968036)
Supplement: Supplementary file 1 [file Data_Sheet_1.docx]

Simultaneous Measurement of the Antibody Responses against SARS-CoV-2 and its Multiple Variants by a Phage Display Mediated Immuno-Multiplex Quantitative PCR Based Assay

Hanyi Chen, Shen Li, Jiali Wang, Siqi He, Dong Wang, Zhaohui Qian, Chenyi Luo, Jianxun Wang

Supplementary Material


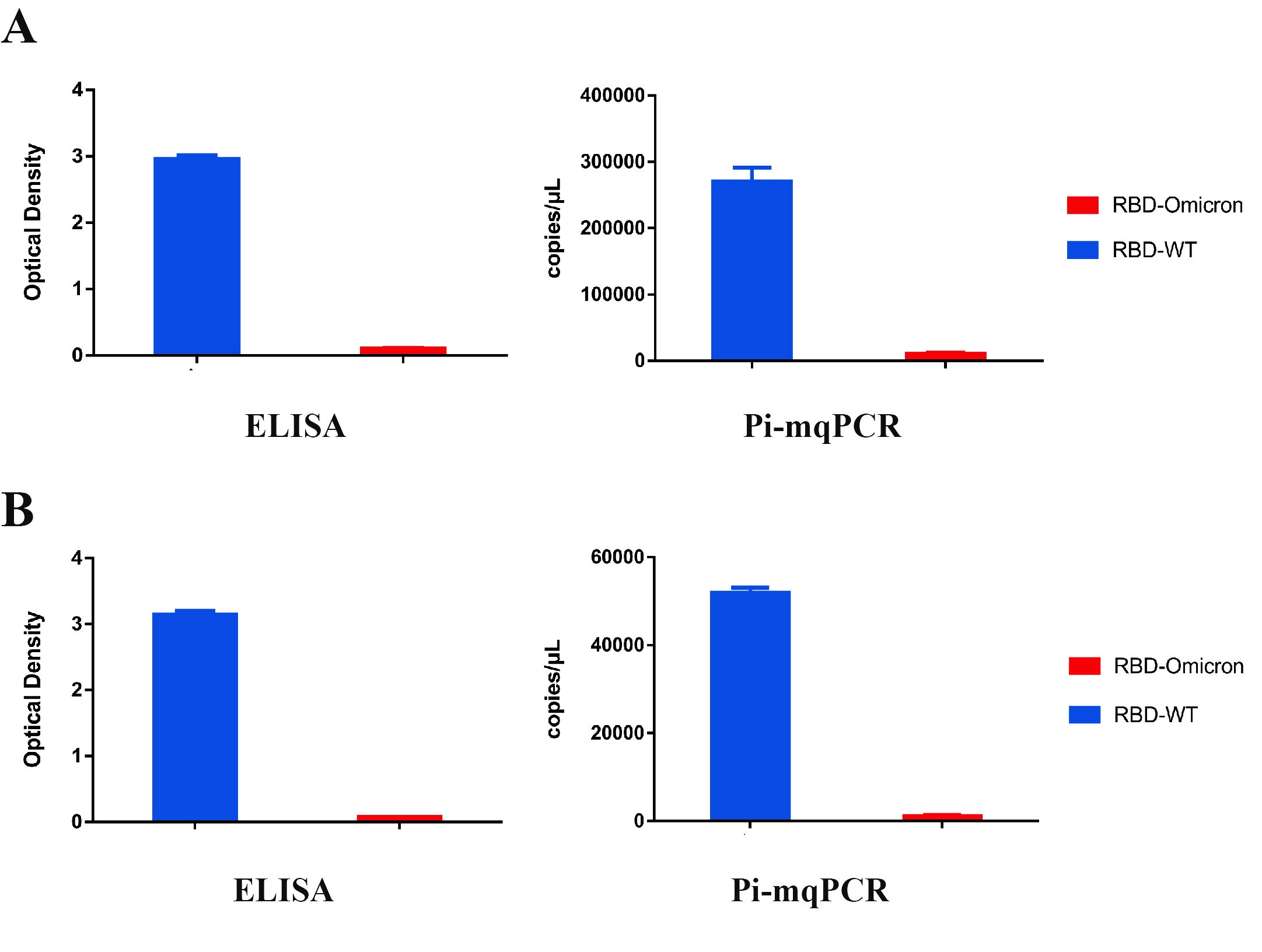


**Supplementary Figure 1.** Comparison of standard ELISA method with Pi-mqPCR method with nanobody N4**(A)** and N6**(B)**.

# Supplementary Data

**The sequences of RBD-coding gene from the wild type of SARS-COV2**

CCGAACATCACCAACCTGTGCCCGTTTGGCGAGGTTTTCAACGCGACCCGTTTCGCGAGCGTGTACGCGTGGAACCGTAAACGTATCAGCAACTGCGTTGCGGACTATAGCGTGCTGTACAACAGCGCGAGCTTCAGCACCTTTAAGTGCTATGGTGTGAGCCCGACCAAACTGAACGATCTGTGCTTTACCAACGTTTACGCGGATAGCTTCGTGATTCGTGGCGACGAGGTTCGTCAGATCGCGCCGGGTCAAACCGGCAAGATTGCGGACTACAACTATAAACTGCCGGACGATTTCACCGGTTGCGTTATCGCGTGGAACAGCAACAACCTGGATAGCAAAGTGGGTGGCAACTACAACTATCTGTACCGTCTGTTTCGTAAGAGCAACCTGAAACCGTTCGAGCGTGACATTAGCACCGAAATCTACCAGGCGGGTAGCACCCCGTGCAACGGTGTTGAGGGCTTTAACTGCTATTTCCCGCTGCAGAGCTACGGCTTCCAACCGACCAACGGTGTGGGCTATCAACCGTACCGTGTGGTTGTGCTGAGCTTTGAACTGCTGCATGCGCCG

**The sequences of RBD-coding gene from the SARS-COV2 with N501Y, E484K mutations**

CCGAACATCACCAACCTGTGCCCGTTTGGCGAGGTTTTCAACGCGACCCGTTTCGCGAGCGTGTACGCGTGGAACCGTAAACGTATCAGCAACTGCGTTGCGGACTATAGCGTGCTGTACAACAGCGCGAGCTTCAGCACCTTTAAGTGCTATGGTGTGAGCCCGACCAAACTGAACGATCTGTGCTTTACCAACGTTTACGCGGATAGCTTCGTGATTCGTGGCGACGAGGTTCGTCAGATCGCGCCGGGTCAAACCGGCAAGATTGCGGACTACAACTATAAACTGCCGGACGATTTCACCGGTTGCGTTATCGCGTGGAACAGCAACAACCTGGATAGCAAAGTGGGTGGCAACTACAACTATCTGTACCGTCTGTTTCGTAAGAGCAACCTGAAACCGTTCGAGCGTGACATTAGCACCGAAATCTACCAGGCGGGTAGCACCCCGTGCAACGGTGTTAAGGGCTTTAACTGCTATTTCCCGCTGCAGAGCTACGGCTTCCAACCGACCTACGGTGTGGGCTATCAACCGTACCGTGTGGTTGTGCTGAGCTTTGAACTGCTGCATGCGCCG

**The sequences of RBD-coding gene from the SARS-COV2 with L452R, T478K mutations (B.1.617.2, delta variant)**

CCGAACATCACCAACCTGTGCCCGTTTGGCGAGGTTTTCAACGCGACCCGTTTCGCGAGCGTGTACGCGTGGAACCGTAAACGTATCAGCAACTGCGTTGCGGACTATAGCGTGCTGTACAACAGCGCGAGCTTCAGCACCTTTAAGTGCTATGGTGTGAGCCCGACCAAACTGAACGATCTGTGCTTTACCAACGTTTACGCGGATAGCTTCGTGATTCGTGGCGACGAGGTTCGTCAGATCGCGCCGGGTCAAACCGGCAAGATTGCGGACTACAACTATAAACTGCCGGACGATTTCACCGGTTGCGTTATCGCGTGGAACAGCAACAACCTGGATAGCAAAGTGGGTGGCAACTACAACTATCGGTACCGTCTGTTTCGTAAGAGCAACCTGAAACCGTTCGAGCGTGACATTAGCACCGAAATCTACCAGGCGGGTAGCAAGCCGTGCAACGGTGTTGAGGGCTTTAACTGCTATTTCCCGCTGCAGAGCTACGGCTTCCAACCGACCAACGGTGTGGGCTATCAACCGTACCGTGTGGTTGTGCTGAGCTTTGAACTGCTGCATGCGCCG

**The sequences of RBD-coding gene from the SARS-COV2 with L452Q, F490S mutations (C37, lambda variant)**

CCGAACATCACCAACCTGTGCCCGTTTGGCGAGGTTTTCAACGCGACCCGTTTCGCGAGCGTGTACGCGTGGAACCGTAAACGTATCAGCAACTGCGTTGCGGACTATAGCGTGCTGTACAACAGCGCGAGCTTCAGCACCTTTAAGTGCTATGGTGTGAGCCCGACCAAACTGAACGATCTGTGCTTTACCAACGTTTACGCGGATAGCTTCGTGATTCGTGGCGACGAGGTTCGTCAGATCGCGCCGGGTCAAACCGGCAAGATTGCGGACTACAACTATAAACTGCCGGACGATTTCACCGGTTGCGTTATCGCGTGGAACAGCAACAACCTGGATAGCAAAGTGGGTGGCAACTACAACTATCAGTACCGTCTGTTTCGTAAGAGCAACCTGAAACCGTTCGAGCGTGACATTAGCACCGAAATCTACCAGGCGGGTAGCACCCCGTGCAACGGTGTTGAGGGCTTTAACTGCTATTCCCCGCTGCAGAGCTACGGCTTCCAACCGACCAACGGTGTGGGCTATCAACCGTACCGTGTGGTTGTGCTGAGCTTTGAACTGCTGCATGCGCCG

**The sequences of RBD-coding gene from the SARS-COV2 with L452R, E484Q mutations (B.1.617.1, kappa variant)**

CCGAACATCACCAACCTGTGCCCGTTTGGCGAGGTTTTCAACGCGACCCGTTTCGCGAGCGTGTACGCGTGGAACCGTAAACGTATCAGCAACTGCGTTGCGGACTATAGCGTGCTGTACAACAGCGCGAGCTTCAGCACCTTTAAGTGCTATGGTGTGAGCCCGACCAAACTGAACGATCTGTGCTTTACCAACGTTTACGCGGATAGCTTCGTGATTCGTGGCGACGAGGTTCGTCAGATCGCGCCGGGTCAAACCGGCAAGATTGCGGACTACAACTATAAACTGCCGGACGATTTCACCGGTTGCGTTATCGCGTGGAACAGCAACAACCTGGATAGCAAAGTGGGTGGCAACTACAACTATCGGTACCGTCTGTTTCGTAAGAGCAACCTGAAACCGTTCGAGCGTGACATTAGCACCGAAATCTACCAGGCGGGTAGCACCCCGTGCAACGGTGTTcAGGGCTTTAACTGCTATTTCCCGCTGCAGAGCTACGGCTTCCAACCGACCAACGGTGTGGGCTATCAACCGTACCGTGTGGTTGTGCTGAGCTTTGAACTGCTGCATGCGCCG

**The sequences of RBD-coding gene from the SARS-COV2 omicron variant (B.1.1.529)**

CCCAATATCACAAACCTGTGCCCTTTTGACGAGGTGTTCAACGCAACCAGGTTCGCAAGCGTGTACGCATGGAATAGGAAGCGCATCTCCAACTGCGTGGCCGACTATTCTGTGCTGTACAACCTGGCCCCCTTCTTCACCTTTAAGTGCTATGGCGTGAGCCCCACAAAGCTGAATGACCTGTGCTTTACCAACGTGTACGCCGATTCCTTCGTGATCAGGGGCGACGAGGTGCGCCAGATCGCACCAGGACAGACAGGCAACATCGCAGACTACAATTATAAGCTGCCTGACGATTTCACCGGCTGCGTGATCGCCTGGAACTCTAACAAGCTGGATAGCAAAGTGAGCGGCAACTACAATTATCTGTACCGGCTGTTTAGAAAGTCTAATCTGAAGCCATTCGAGAGGGACATCTCCACAGAGATCTACCAGGCCGGCAACAAGCCCTGCAATGGCGTGGCCGGCTTTAACTGTTATTTCCCTCTGCGGAGCTACAGCTTCCGGCCAACCTACGGCGTGGGCCACCAGCCCTACCGCGTGGTGGTGCTGTCTTTTGAGCTGCTGCACGCACCT
